# Shared Decision Making and Cardioneuroablation Allow Discontinuation of Permanent Pacing in Patients with Vagally Mediated Bradycardia

**DOI:** 10.3390/jcdd10090392

**Published:** 2023-09-11

**Authors:** Sebastian Stec, Antoni Wileczek, Agnieszka Reichert, Janusz Śledź, Jarosław Kosior, Dariusz Jagielski, Anna Polewczyk, Magdalena Zając, Andrzej Kutarski, Dariusz Karbarz, Dorota Zyśko, Łukasz Nowarski, Edyta Stodółkiewicz-Nowarska

**Affiliations:** 1 Division of Electrophysiology, Cardioneuroablation, Catheter Ablation and Cardiac Stimulation, Subcarpathian Center for Cardiovascular Intervention, 38-500 Sanok, Poland; 2 Department of Invasive Cardiology, County Specialistic Hospital, 37-450 Stalowa Wola, Poland; 3 El-Medica, EP-NETWORK, 26-110 Skarzysko-Kamienna, Poland; 4 Department of Cardiology, Masovian Specialist Hospital, 26-617 Radom, Poland; 5 Department of Cardiology, Centre for Heart Diseases, 4th Military Hospital, 50-981 Wroclaw, Poland; 6 Faculty of Medicine, Wrocław University of Science and Technology, 50-370 Wroclaw, Poland; 7 Department of Physiology, Pathophysiology and Clinical Immunology, Institute of Medical Sciences, Jan Kochanowski University, 25-369 Kielce, Poland; 8 Department of Cardiac Surgery, Świętokrzyskie Center of Cardiology, 25-736 Kielce, Poland; 9 Department of Special Pedagogy and Speech Therapy, Kazimierz Wielki University, 85-064 Bydgoszcz, Poland; 10 Department of Cardiology, Medical University, 20-059 Lublin, Poland; 11 Department of Emergency Medicine, Wrocław Medical University, 50-367 Wroclaw, Poland; 12 Vascular Surgery Department, The Brothers of Saint John of God Hospital, 31-061 Krakow, Poland; 13 Institute of Cardiovascular Science, 31-008 Krakow, Poland

**Keywords:** bradycardia, cardioneuroablation, syncope, pacemaker, transvenous lead extraction, autonomic tests, shared decision making

## Abstract

Background: Safe discontinuation of pacemaker therapy for vagally mediated bradycardia is a dilemma. The aim of the study was to present the outcomes of a proposed diagnostic and therapeutic process aimed at discontinuing or not restoring pacemaker therapy (PPM) in patients with vagally mediated bradycardia. Methods: The study group consisted of two subgroups of patients with suspected vagally mediated bradycardia who were considered to have PPM discontinued or not to restore their PPM if cardioneuroablation (CNA) would successfully treat their bradycardia. A group of 3 patients had just their pacemaker explanted but reimplantation was suggested, and 17 patients had preexisting pacemakers implanted. An invasive electrophysiology study was performed. If EPS was negative, extracardiac vagal nerve stimulation (ECVS) was performed. Then, patients with positive ECVS received CNA. Patients with an implanted pacemaker had it programmed to pace at the lowest possible rate. After the observational period and control EPS including ECVS, redo-CNA was performed if pauses were induced. The decision to explant the pacemaker was obtained based on shared decision making (SDM). RESULTS: After initial clinical and electrophysiological evaluation, 17 patients were deemed eligible for CNA (which was then performed). During the observational period after the initial CNA, all 17 patients were clinically asymptomatic. The subsequent invasive evaluation with ECVS resulted in pause induction in seven (41%) patients, and these patients underwent redo-CNA. Then, SDM resulted in the discontinuation of pacemaker therapy or a decision to not perform pacemaker reimplantation in all the patients after CAN. The pacemaker was explanted in 12 patients post-CNA, while in 2 patients explantation was postponed. During a median follow-up of 18 (IQR: 8–22) months, recurrent syncope did not occur in the CNA recipients. Conclusions: Pacemaker therapy in patients with vagally mediated bradycardia could be discontinued safely after CNA.

## 1. Introduction

The management of sinus bradycardia or atrioventricular block (AVB) is often challenging and requires a comprehensive diagnostic workup. Symptomatic sinus node dysfunction, atrioventricular block (AVB), cardioinhibitory vasovagal syncope (VVS), or carotid sinus syndrome could be vagally mediated and pacemaker implantation is their treatment option [1]. However, lifelong cardiac implantable electronic devices are associated with a risk of cardiovascular complications, a need for a multiple generator (and possible lead) replacement, and impose certain restrictions on professional or sports activities. Therefore, the current guidelines and expert opinion statements recommend that each individual patient with a pacemaker should be reassessed prior to subsequent device replacement or discontinuation of pacing therapy [1,2,3,4,5,6,7]. However, there is currently no specific standardized protocol or approach using shared decision making to assess such patients [7,8,9]. The implementation of novel diagnostic and therapeutic modalities, extracardiac vagal nerve stimulation (ECVS), and anti-bradycardia therapy via percutaneous catheter-based cardioneuroablation (CNA), may enable discontinuation of pacemaker therapy or pacemaker explantation after shared decision making. These interventions are associated with high clinical success rates [1,8,9,10,11].

Vagally mediated sinus bradycardia and bradycardia due to vagally mediated atrio-ventricular block may resolve after atropine administration [12]. An increase in heart rate after administration of atropine is considered evidence of the presence of tonic vagal activity whereas lack of an increase indicates intrinsic, non-vagal, bradycardia. Acceleration of the heart rate/prevention of bradycardia incidents can be obtained by damaging the peripheral postgangionated vagus nerve arch.

The purpose of this study was to present the outcomes of a diagnostic and therapeutic modality aimed at discontinuing, or not restoring, pacemaker therapy in patients with vagally mediated bradycardia.

## 2. Material and Methods

The study was designated as a prospective observational study of a subset of patients registered into the Rare-A-CaREgistry in whom CNA and explantation of the previously implanted pacemaker was under consideration. In addition, patients whose pacemaker was explanted during the previous 12 months and had recurrent syncope (in whom CNA was planned) were included in the study.

Rare-A-CaREgistry was approved by the local institutional ethics committee (Rzeszow University, decision number 5/4/2017-RARE-A-CAREgistry) and was conducted in accordance with the Declaration of Helsinki. The registry covered ablation and CNA procedures performed at 10 Polish electrophysiology (EP) centers by the same team of 3 experienced operators and EP fellows in training.

All patients provided written informed consent for the procedure and protocol tests. The patients were informed that the CNA is a relatively new method not recommended by the current guidelines, but the personal experience of the researchers indicates its usefulness.

The enrollment of the patients with presumed vagally mediated bradycardia relied on the patients’ willingness to have their pacemaker explanted and the increase in the heart rate by more than 30% after administration of 0.02–0.04 mg/kg i.v. atropine. The atropine tests were done by their attending doctors who considered them candidates for referral for CNA.

The second indication for the CNA was the presence of permanent bradycardia or atrio-ventricular block or the presence of temporary cardioinhibition during noninvasive or invasive autonomic testing. If pacing indications were not confirmed during further testing, the patient could have his pacemaker explanted without performing a second CNA.

The exclusion criterion for CNA was the presence of the prolonged HV interval >70 ms or infranodal AVB during electrophysiological study (EPS).

Shared decision making (SDM) was used in all patients and consisted of informed consent to the CNA and transvenous lead extraction (TLE) procedures and possible alternative treatments, with a discussion regarding outcomes, complications, and possible further actions. Before TLE, all patients were assessed using SAFeTY-TLE score. All this information was presented at the enrollment of patients, with the intention to provide information about the whole diagnostic and therapeutic process.

### 2.1. Eligible Patients Selection

The detailed medical history, the primary indication for pacemaker implantation, and ECG documentation of sinus bradycardia or AVB were reviewed. The use of drugs that might influence sinoatrial (SA) and atrioventricular (AV) node function were also evaluated. Additionally, if an organic disease was suspected, computed tomography or magnetic resonance imaging were recommended. Ultrasonography of the neck was performed to exclude venous thrombosis as well as structural diseases of the neck and subcranial regions. Assessments of pacemaker function and the need for pacing were evaluated noninvasively.

### 2.2. EPS

EPS was performed with 2 catheters. A decapolar catheter was introduced into the coronary sinus and a quadripolar steerable catheter was positioned to record a His bundle electrogram. Standard parameters of SA and AV nodes were assessed. Special attention was paid to the validation of proximal (intranodal) and distal (infranodal) AV block with a detailed His bundle electrogram recorded at baseline and during programmed and incremental atrial pacing. The presence of an HV interval longer than 70 ms, or infranodal AVB during incremental atrial pacing was considered evidence of abnormal AVB. In some patients, EPS or noninvasive EPS was performed as a separate procedure to confirm the efficacy of atropine on atrioventricular nodal properties in the final shared decision-making process. Improvement in AV conduction after atropine administration was deemed a predictor of CNA efficacy.

### 2.3. ECVS

Before CNA, patients were sedated, intubated, and studied under general anesthesia. The muscles were relaxed using mivacurium chloride or succinylcholine. Next, ECVS from the right internal jugular vein was performed during spontaneous sinus rhythm and proximal coronary sinus pacing to confirm prolonged sinus node recovery and/or AVB. ECVS included the evaluation of the subcranial and cervical course of the internal jugular vein. At least unilateral ECVS was performed at baseline. Bilateral ECVS was attempted but waived after unsuccessfully trying for 10 min, in 2 patients. A quadripolar irrigated electrode was advanced via the superior vena cava and internal jugular vein, up to the jugular foramen. A portable ultrasound system (Butterfly IQ+, Butterfly Network, Guilford, CT, USA) was used to guide a tip catheter position in proximity to the vagal nerve course during ECVS. ECVS was performed by a pulsed electric field (pulse amplitude of 1 V/kg body weight up to 70 V, 50 µsec width, 50 Hz frequency during 5 s) within the subcranial region of the internal jugular vein according to Pachon’s methodology [13,14,15,16]. The primary goal of CNA was to achieve sinus and atrioventricular node parasympathetic denervation that resulted in a post-ECVS absence of sinus arrest and advanced AVB with bradycardia. If ECVS-induced sinus bradycardia or advanced AVB persisted, further applications with radiofrequency were performed in the region with fragmented signals and paced or ablation-induced reflex bradycardia. The final ECVS was performed 15 min after the last application [13,14].

### 2.4. CNA

After baseline EPS and ECVS, step-by-step biatrial endocardial ablation at 6 anatomic sites of the ganglionated plexi (GP) mapped according to the modified Pachon’s method was performed in the left and right atria [12]. Single or double (in patients with atrial fibrillation and a need for pulmonary vein isolation) transseptal puncture was obtained to achieve left atrial access. A 3-dimensional electroanatomic system was used to delineate the left atrial contour. A 4 mm irrigated catheter (Alcath, Biotronik, Berlin, Germany) was advanced into the left atrium and each pulmonary vein to create a left atrial map. Six anatomically guided areas of presumed GP were ablated. Usually, while waiting for muscle relaxation effects to disappear, the left-sided GPs were ablated first with the monitoring of the Wenckebach point, AVN ERP (measured as S1–S2 ERP), and sinus rhythm rate. In patients with carotid sinus hypersensitivity or carotid sinus syndrome, carotid sinus massage was applied to find the most valuable GP or the site for denervation and resolution of bradycardia induced by carotid sinus massage. Before ablation of the right-sided pulmonary veins, the superior vena cava, and the septal site of the superior vena cava—right atrial junction—pacing (25 mA at 2.0 ms pulse width at a cycle length of 600 ms) was performed from the ablation catheter to localize the position of the right phrenic nerve before and during ablation. Before ablation in close proximity to the sinus node, the position of the sinus node was mapped and localized by determining the earliest local activation time and recording a negative unipolar electrogram preceding by 20–30 ms the beginning of the P wave on surface ECG. Biatrial, binodal CNA with complete resolution of sinus pauses and advanced AVBs during bilateral ECVS was the endpoint of the procedure. Intracardiac signals were recorded using the EP-TRACER system (Schwarzer Cardiotek GmbH, GL Sittard, The Netherlands). Catheter navigation and mapping were facilitated by a 3D electroanatomic system (EnSite NavX/Precision™; Abbott, St. Paul, MN, USA). Finally, intravenous atropine was administered to confirm the binodal efficacy of CNA, the absence of a significant increase in sinus rhythm, and the improvement of parameters of atrioventricular node conduction. It is expected that successful CNA disables heart rate increase after atropine administration because there is no parasympathetic tone that could be suppressed by atropine.

### 2.5. Additional Substrate Ablation

In patients with additional indications for catheter ablation, substrate modification was planned either for supraventricular or ventricular arrhythmias if necessary.

### 2.6. Re-Evaluation Strategy before Discontinuation of Pacemaker Therapy

Noninvasive EPS, device control, and pacing burden (with VVI or AAI mode programmed at 30 bpm and prolonged ECG monitoring) were assessed to confirm the disappearance of bradycardia and the need for pacing. Subsequently, patients were referred for control EPS including obligatory ECVS, and, if needed, additional redo-CNA. If only a single area of repeat GP ablation was required, the patient was referred for TLE or further noninvasive testing.

The first case accepted for discontinuation of pacemaker therapy after CNA was referred in May 2019. The final decision on the discontinuation of pacemaker therapy was based on shared decision making that encompassed several aspects of arrhythmia management, risk-benefit assessment, patient-related outcomes, and autonomy.

### 2.7. Pacemaker Explantation

The term pacing system explantation is used for generator explantation and transvenous lead extraction (TLE). The risk related to the procedure of pacemaker explantation is related mainly to the TLE explantation [6,7,17,18]. The procedures were conducted in a hybrid operating theatre in a cardiac surgery department, where all the necessary equipment and additional staff were available in case a rescue intervention was needed. The risk of major complications related to TLE was assessed using the SAFeTY TLE score as low, intermediate, and very high [19]. Lead extraction procedures were performed using mechanical systems such as polypropylene Byrd dilator sheaths (Cook^®^ Medical, Leechburg, PA, USA), mainly via the subclavian approach on the side of the implanted device. In case of technical difficulties, additional vascular access and/or additional tools were applied [Evolution (Cook^®^ Medical, Bloomington, IN, USA), TightRail (Spectranetix, Colorado Springs, CO, USA), lassos, and basket catheters] [17,18,20]. Laser-cutting sheaths were not used. All TLEs were performed by the same experienced pacemaker explantation operator and another operator with experience in pacing therapy. Additionally, a cardiac surgeon, an anesthesiologist, and an echocardiographer were present in most high-risk patients.

### 2.8. Statistical Analysis

The continuous variables were presented as median, range, and interquartile range (IQR). The discrete variables were presented as numbers and percentages. The final follow-up was performed in December 2022.

## 3. Results

Between June 2018 and January 2022, we enrolled 20 patients with suspected vagally mediated bradycardia, who were candidates to discontinue pacemaker therapy (if CNA was performed successfully), in the study. The study group consisted of two subgroups of patients: a subgroup of 17 patients who had an implanted pacemaker and a subgroup of 3 patients who had just had the pacemaker explanted but reimplantation was suggested. In the latter patients, the reason for CNA referrals was fear of recurrent syncope due to paroxysmal AVB (after lead-related endocarditis) in one patient, and syncopal relapse in two patients.

### 3.1. Demographics and Previous Pacemaker Therapy

The demographics, clinical characteristics, index arrhythmias, and pacemaker therapy details are presented in Table 1. The study group included 8 women and 12 men with median age of 38.0 (IQR 34.5–40.0) years. Previous TLE or generator replacement were reported in three and seven of these patients, respectively, with continuation of pacemaker therapy. The median time of pacemaker therapy was 68.5 (IQR 32.5–156.5) months (range: 2–372). Eighteen (90%) patients were in the 18–65 age group. Three patients, so-called urgent referrals, were assessed after TLE was performed for various reasons, notably less than one month before enrolment—but at the time of initial assessment, these patients did not have implanted pacemakers.

### 3.2. Electrophysiology Study, CNA Qualification, and Short-Term Results

The details of the baseline electrophysiology study and ECVS are presented in Table 2. All the studied patients had a sinus pause during baseline ECVS. Baseline ECVS performed during atrial pacing also resulted in advanced/complete AVB in all the patients.

CNA was not performed after ECVS and EPS in 3 of 20 patients. The first patient had persistent structural advanced second- and third-degree AVB, and was indicated for TLE and His-bundle pacing upgrade. The second patient had a history of incidental persistent third-degree AVB more than 15 years earlier without any further bradycardia episodes and he was referred for pacemaker explantation and TLE. Finally, the third patient had incidental sinus node dysfunction or tachycardia-bradycardia syndrome, and a decision was made to continue pacemaker therapy until elective replacement indication (Table 2).

In the remaining 17 cases, CNA was performed. The median duration of the procedure was 95 IQR 75–123 min (range: 60–210). The studied electrophysiologic parameters after index CNA are presented in Table 2. During ECVS after CNA, there were no pauses.

### 3.3. Additional Substrate Ablation

The ablation of additional paroxysmal tachycardias was performed in two patients: one with paroxysmal atrial fibrillation and one with atrial flutter.

### 3.4. Clinical Evaluation after CNA

During follow-up, there were no syncopal or presyncopal events. All the patients with implanted pacemakers had <1% paced QRS complexes reported by device interrogation. A subgroup of seven (41.1%) otherwise asymptomatic patients during control EPS with ECVS had sinus pause or atrioventricular block. CNA was repeated in these patients during the same procedure.

### 3.5. Shared Decision to Explant Pacemaker

The recommendation to discontinue pacemaker therapy was primarily considered in 17 patients. SDM resulted in pacemaker therapy being continued in three patients, including: (1) A patient with recurrent syncope despite pacemaker therapy (complex syncope with VVS and psychogenic pseudosyncope), who refused further investigation and a monitored rehabilitation program, (2) An elderly patient with advanced ischemic heart disease,(3) A young patient with incidental asymptomatic 4.5 s AVB during ILR monitoring prior and after second CNA, generator replacement, no TLE performed.

### 3.6. Pacemaker Explantation and the Risk of TLE Procedures

Shared decision making led to the resignation from permanent pacing in 19 (age range: 18–57) of 20 cases (95%). Summarizing the data, 14 (70%) of the whole group of 20 patients had CNA followed by discontinuation of pacemaker therapy. Moreover, one patient had excluded indications for CNA and the necessity for permanent pacing, thus no ECVS or CNA were performed. Ultimately, after shared decision making, discontinuation of permanent pacemaker therapy, including the decision to explant and the decision not to reimplant, occurred in 17 (75%) out of 20 patients. A total of 3 out of 19 (15.7%) patients, after SDM decided to discontinue pacemaker therapy and extract only at early replacement indicated (ERI).

The median SAFeTY TLE score for the whole group was 1.52 (IQR 0.31–2.98). On the basis of the SAFeTY TLE score, the probability of major complications was low in six patients, intermediate in four patients, and high or very high in six patients. Despite the significant risk, no serious complications were associated with invasive treatment and TLE. The study also revealed the presence of obstacles in using the effects of the successful index CNA for pacing discontinuation due to the high rate of ECVS-induced pauses in control EPS. The details of pacemaker explantation procedures are presented in Table 3.

Experts’ opinions of the possibility of discontinuation of PPM therapy were in close relation to the conducted evaluation of CNA efficacy (Table 4) (Chi2 = 20.0; df = 3; *p* = 0.00017). Similarly, SDM to explant/not to reimplant was dependent on prior electrophysiological evaluation (Chi2 = 14.44; df = 3, *p* = 0.00236) and expert opinion (Chi2 = 9.473; df = 1; *p* = 0.00208). In addition, expert opinions on the possibility of PM explantation were not observed to be related to the risk of complications (Chi2 = 3.15, df = 3; *p* = 0.3679), at a slightly higher level of trend statistics related to the relationship of SDM and the risk of complications (Chi2 = 6.66, df = 3; *p* = 0.08332). However, when comparing the number of explantations in those undertaking SDM, a higher number of approvals for the procedure is apparent (chi2 McNemar = 11.52; df = 1, *p* = 0.0007).

**Figure 1:**
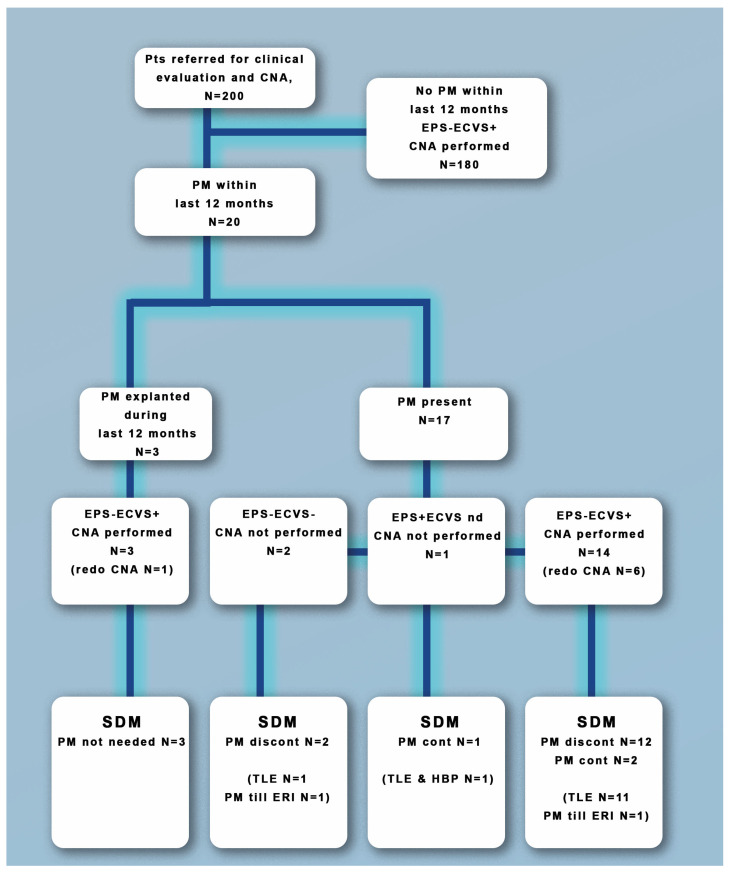
The flow of the patients referred for cardioneuroablation. CNA: cardioneuroablation. ECVS+: the presence of a pause during vagal nerve stimulation. ECVS−: lack of a pause during vagal nerve stimulation. ECVS: extracardiac vagal stimulation. EPS: electrophysiological study. EPS+: HV > 70 ms or distal AV block during incremental pacing. EPS-: HV < 70 ms and lack of distal AV block during incremental pacing. n.d.: not done. PM: pacemaker therapy. PM cont: pacemaker therapy continuation. PM discount: pacemaker therapy discontinuation. SDM: shared decision making. TLE: transvenous lead extraction.

**Figure 2:**
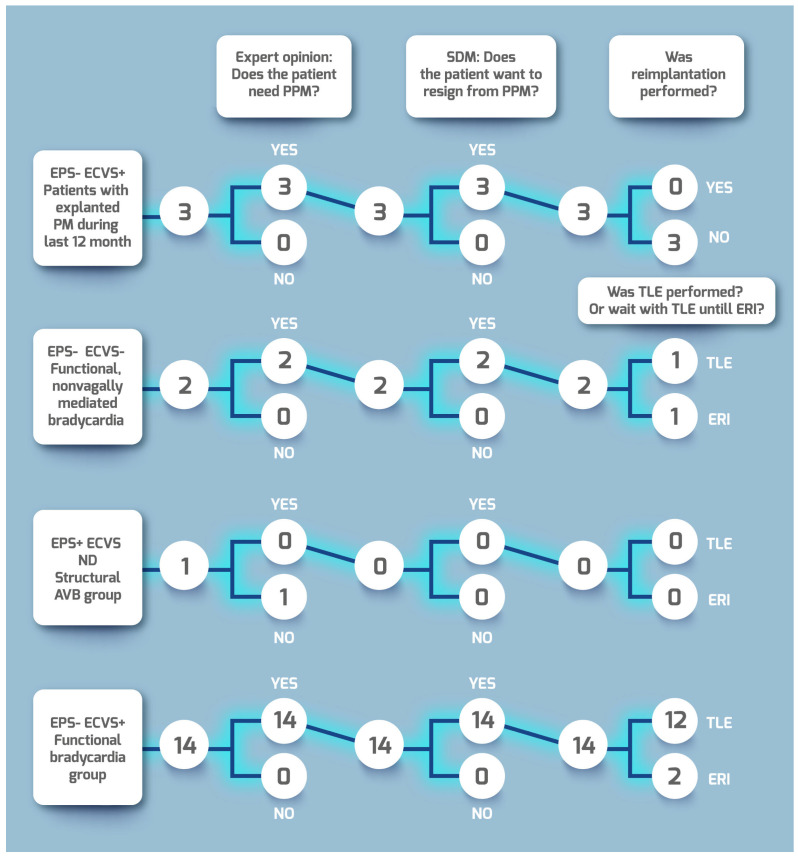
Line graph detailing the patient flow.

This means that patients participating in SDM who are comprehensively presented with the benefits and possible complications make a rational, calculated treatment decision. Patient flow related to EPS, ECVS and CNA is is clearly presented on Figure 1. Patient flow related to Experts’ opinion, SDM and TLE is presented on Figure 2.

### 3.7. Follow-Up after Pacemaker Explantation

During the median of 18 months (IQR 8–22) after the pacemaker explantation and discontinuation of pacemaker therapy, no patients reported syncope, presyncope, severe fatigue, or palpitation. One woman became pregnant and delivered a premature, otherwise healthy, infant.

## 4. Discussion

This study presents an approach that allows discontinuation of pacemaker therapy in most patients with vagally mediated bradycardia. This approach has resulted in no complications at the midterm follow-up and should be considered safe by physicians and patients.

Several centers perform CNA, either following the original methodology or with modifications [19,21,22,23,24,25,26,27,28]. In our opinion, the methodological problem of establishing endpoints for CNA trials was resolved by the implementation of ECVS by Pachon et al. [10]. The ECVS procedure enables the assessment of vagal nerve influence on sinus bradycardia and AV conduction and makes it possible to establish a clear endpoint for the CNA procedure [12]. In our study, ECVS was used for the first time to validate not only the efficacy of baseline, index CNA procedure but also during follow-up to enable a final decision on additional CNA, TLE, and/or discontinuation of pacemaker therapy. Using control ECVS 2–4 months after baseline during control EPS procedure resulted in additional CNA in about 40% of otherwise asymptomatic patients. Therefore, the final decision was made on the second negative invasive ECVS or negative ECVS directly at the end of the redo-CNA.

A recent multi-center pilot study was the first to confirm the feasibility and high efficacy of fragmented ECG–guided GP ablation across a large group of procedure-naive operators. Although a sizable number of patients did not report recurrent syncope during follow-up, there was no strict ECG monitoring or noninvasive/invasive testing to confirm complete persistent cardioneuromodulation during follow-up. In an international survey, most respondents (79%) would opt to refer a patient with refractory VVS for CNA [26]. Although the majority would recommend biatrial CNA with an anatomical approach in combination with autonomic stimulation, there is a lack of data for patients with a pacemaker regardless of the functional etiology of bradycardia, perhaps raising some additional questions and pointing to the need for more comprehensive strategies. One of the largest and most important studies was conducted in Brazil in 2002 [8]. It showed that 35 of the 70 patients (50%) without precise indications for pacemaker therapy could undergo device explantation. Patients underwent clinical and neurological assessment, echocardiography, exercise testing, tilt table test with further reprogramming to the lowest pacing values, and ECG monitoring. Patients who were asymptomatic after 12 months of monitoring underwent an EPS, while those with VVS could not undergo explantation according to the study protocol [8]. Our study showed that with the use of autonomic tests and noninvasive and invasive EPS, ECVS, and control EPS, with obligatory ECVS and redo-CNA if necessary, the time to final discontinuation of pacemaker therapy can be shortened to 4–6 months. However, the use of implantable loop recorders after CNA would confirm the effectiveness of the treatment with certainty.

In the literature, there are sparse data on the management of patients who had pacemaker therapy discontinuation after CNA. Single cases of patients were reported in whom pacing was discontinued after CNA as a result of bradycardia treatment, complications of pacemaker therapy, or when other etiologies of a transient loss of consciousness were misdiagnosed [13,14,29]. Moreover, a national Danish survey including young adults (age < 50 years) with pacemakers and AVBs demonstrated that the incidences of reflex and unexplained etiology were 5% and 50%, respectively [30,31]. Shared decision making may be particularly relevant in young patients when the initial indications for pacemaker implantation were imprecise or appear to have resolved (e.g., transient heart block during an infection), or when control EPS including ECVS confirmed the efficacy of CNA [1,2,3,4,5,6,7,8]. There is only one registered study that evaluates prolonged ECG monitoring by implantable loop recorders, and there are no studies designed to perform invasive evaluation of the efficacy of CNA [22,32]. Therefore, it is necessary to account for the different individual scenarios, to apply approaches based on interdisciplinary cooperation, and to develop patient-tailored management plans. In patients who underwent implantation at a young age with a long dwelling time, pacemaker therapy is associated with a higher risk of complications (such as re-implantations, infections, and reduced quality of life) and a higher risk of TLE procedures. The complexity of the procedures (e.g., noninvasive testing, invasive EPS, ECVS, pacemaker replacement, TLE, prolonged ECG monitoring), together with the inherent risks and limitations, necessitates the interdisciplinary approach with individualized shared decision making. Interdisciplinary cooperation and repeated follow-up are warranted, especially when different clinical scenarios are possible in the modern and rapidly developing era of electrophysiology and CNA [7,8].

Patients with pacemakers implanted due to vasovagal syncope are usually young. A prolonged duration of pacemaker therapy may inevitably subject these individuals to an increased risk of complications. Therefore, their management may result in great dilemmas for physicians involved in their care.

The approach to discontinuing pacemaker therapy in these patients is evolving. We believe that the process of creating guidelines should be preceded by studies presenting results of alternative solutions to this problem.

## 5. Conclusions

CNA is a rapidly developing technique associated with high efficacy and a low periprocedural risk of complications. Its introduction by Jose Carlos Pachon et al. has significantly changed the treatment strategy for patients with VVS and recurrent syncope [7,10,15,16]. Because multicenter randomized controlled trials and standardized endpoints are lacking, this remains an experimental technique. International randomized studies are needed prior to recommending this procedure to treat patients with a wide range of functional bradycardia, including sinus node dysfunction and AVB [13,14,15,16,19,21,23,24,25,26,27,29,32,33,34]. During the course of this study, we implemented EPS and ECVS to qualify patients for index CNA, used ECVS immediately after index CNA to assess its success, and pursued a strategy of obligatory invasive control after 2–4 months; as all patients had control EPS with ECVS, it sometimes led to redo-CNA, again, acutely assessed by ECVS for effect. On this basis, we developed a structured, repetitive approach for future use and demonstrated a lack of middle-term complications.

## 6. Limitations

This study has several limitations. First, the study group is small and the follow-up is relatively short. The long-term clinical outcome of CNA may be affected by incomplete ablation and consequent reinnervation by failure to eliminate intramural parasympathetic postganglionic neurons. The risk of developing bradycardia of a different etiology should be considered. The gold standard of rhythm monitoring is use of an implantable loop recorder which was only employed in two patients in the present group. Furthermore, studies are needed to determine the appropriate timing of noninvasive tests as well as EPS and ECVS after CNA, especially in asymptomatic patients with ECVS-guided clinical decision making during control tests. However, concerns about the patients’ willingness to undergo pacemaker explantation and their physicians’ conviction about the safety, satisfaction, and benefits of avoiding subsequent replacements spur the need to expand knowledge in this field.

It should be stressed that treating bradycardia with vagal denervation does not restore normal heart rate variability but prevents heart rate decelerations often at the cost of sinus tachycardia. It is not known whether the adequate acceleration of the heart rate after atropine excludes intrinsic bradycardia and provides protection against bradycardia especially when caused by AVB.

## Figures and Tables

**Table 1:** Demographics, clinical characteristics, index arrhythmias, and pacemaker (PM) therapy details.

		PM Explanted during the Last 12 Months	Current PM Therapy		
Variable		EPS (−) ECVS (+) N = 3	EPS (−) ECVS (−) N = 2	EPS (+) ECVS ND N = 1	EPS (−) ECVS (+) N = 14
Age, median (IQR)		38 (34–65)	50 (40–60)	39	38 (29–39)
Male gender n (%)		0 (0)	2 (100)	0 (0)	11 (79)
Duration of PM therapy (months)		50 (2–204)	151 (242)	121	68.5 (16–120)
Duration of PM untill discontinuation (months)		85 (2–204)	NA	NA	84 (40–156)
The main indication for PPM	VVS cardioinhibitory				10 (86)
	AVB paroxysmalis	1 (1)			2 (7)
	AVB persistent		1 (50)	1 (100)	
	SND	2 (67)	1 (50)		2 (7)
Symptoms prior to PM therapy Syncope		3 (100)			12 (86)
Symptoms prior to PM therapy Presyncope		3 (100)	1 (50)	1 (100)	13 (93)
Symptoms prior to PM therapy Fatigue		2 (67)	1 (50)	1 (100)	6 (43)
Symptoms prior to PM therapy Palpitation		2 (67)	1 (50)		4 (29)
PM mode		DDD 3 (100)	DDD 2 (100)	DDD 1 (100)	DDD 9 (64) VVI 2 (14) VVi epi 1 (7) VDD (1) AAI (1)

AVB—atrioventricular bloc, SND—Sinus node dysfunction, VVS—vasovagal syncope, PM—pacemaker. Pacing mode DDD—dual chamber pacing, AAI—atrial demand pacing, VVI—ventricular demand pacing, VVI epi—epicardial ventricular demand pacing.

**Table 2:** The details of the baseline electrophysiology study (EPS) and extracardiac vagal stimulation (ECVS) before (b) and after (a) cardioneuroablation.

	PM Explanted during the Last 12 Months	Current PM Therapy		
Variable	EPS (−) ECVS (+) N = 3	EPS (−) ECVS (−) N = 2	EPS (+) ECVS ND N = 1	EPS (−) ECVS (+) N = 14
SNRTb (ms)	853 (700–960)	1075 (900–1250)	2000	990 (950–1200)
cSNRTb (ms)	220 (100–360)	390 (360–420)	900	25 (210–400)
HVb (ms)	50 (50–54)	54 (50–58)	120	45 (45–50)
PWb (ms)	420 (370–440)	40 (400–400)	900	400 (360–450)
ECVSb [sinus pause], (s)	8.4 (7.0–9.2)	NA	NA	8.15 (6.1–10.5)
ECVSb [avb during A pacing], (s)	8.1 (6.6–9.6)	NA	NA	7.95 (5.2–9.6)
ECVSa (after “last” CNA ^1^) ^2^ (s)	-	ND	ND	-
ECVSa (after “last” CNA ^1^) ^2^ (s)	-	ND	ND	-
SNRTa (ms)	800 (650–890)	ND	ND	710 (670–750)
cSNRTa (ms)	150 (100–280)	ND	ND	210 (180–290)
HVa (ms)	50 (50–54)	ND	ND	45 (45–50)
PWa (ms)	370 (350–390)	ND	ND	360 (320–400)

cSNRT—corrected sinus node recovery time. PM—pacemaker; AVB—atrioventricular block; SNRT—sinus node recovery time, HV—His-ventricle interval. Remarks: ^1^ last CNA—first CNA in patients with one procedure or second CNA in patients with redo-CAN; ^2^ Two patients had delayed TLE due to extensive redo-CNA and invasive control with ECVS.

**Table 3:** Discontinuation of PM therapy and TLE procedures.

	PM Explanted during the Last 12 Months	Current PPM		
Variable	EPS (−) ECVS (+) N = 3	EPS (−) ECVS (−) N = 2	EPS (+) ECVS ND N = 1	EPS (−) ECVS (+) N = 14
Expert opinion: possible PM explantation or no need to reimplant PM (n)	3 (100)	2(100)	0 (0)	14 (100)
SDM to explant/not to reimplant PM (n)	3 (100)	2 (100)	0 (0)	14 (100)
PM explanations (n)	0 (0)	1 (50)	0 (0)	12 (84)
The mean number of previous PM implantations per patient	2.3	1.5	2	1.5
The mean number of previous TLE per patient	1	0	0	0.14
The mean number of previous CIED procedures per patient	1.6	0.5	0	0.8
Safety of TLE Score median (IQR)	1 (0–4)	1.19 (0.16–2.22)	0.41	0.96 (0.28–3.23)
Probability of complications				
Very high (VH)				1
High (H)	2			3
Intermediate (I)		1		3
Low (L)	1	1	1	7

Probability of complications: L—low; I—intermediate; H—high, VH—very high. CIED Cardiac Implantable Electronic Device. PM—pacemaker. SDM—shared decision making. TLE—transcutaneous lead extraction.

**Table 4:** SDM to explant/not to reimplant and explantation PM.

SDM	SDM and Explantation (N/%)		
	Explantation No	Explantation Yes	Total
yes	6/30% *	12/60%	18/90%
no	2/10%	0	2/10%
total	8/40%	12/60%	20/100%

* This table and analysis assume that those who had their PM removed and made a “not to reimplant” decision by the end of the study remained in the explant non-decision group, allowing for a conservative and reliable estimate of the distribution relationship.

## Data Availability

The data that support the findings of this study are available from the corresponding author (S.S.) upon request.
